# Shuang-Huang-Lian Attenuates Airway Hyperresponsiveness and Inflammation in a Shrimp Protein-Induced Murine Asthma Model

**DOI:** 10.1155/2019/4827342

**Published:** 2019-01-01

**Authors:** Yuan Gao, Qiaoling Fei, Ruijuan Qi, Rui Hou, Yixin Han, Runlan Cai, Guibo Sun, Yun Qi

**Affiliations:** Institute of Medicinal Plant Development, Chinese Academy of Medical Sciences & Peking Union Medical College, Beijing 100193, China

## Abstract

Shuang-Huang-Lian (SHL), an herbal formula of traditional Chinese medicine, is clinically used for bronchial asthma treatment. Our previous study found that SHL prevented basophil activation to suppress Th2 immunity and stabilized mast cells through activating its mitochondrial calcium uniporter. Sporadic clinical reports that SHL was used for the treatment of bronchial asthma can be found. Thus, in this study, we systematically investigated the effects of SHL on asthmatic responses using a shrimp protein (SP)- induced mouse model. SHL significantly inhibited airway inspiratory and expiratory resistance, and histological studies suggested it reduced thickness of airway smooth muscle and infiltration of inflammation cells. It also could alleviate eosinophilic airway inflammation (EAI), including reducing the number of eosinophils and decreasing eotaxin and eosinophil peroxidase levels in the bronchoalveolar lavage fluid (BALF). Further studies indicated that SHL suppressed SP-elevated mouse mast cell protease-1 and IgE levels, prevented Th2 differentiation in mediastinal lymph nodes, and lowered Th2 cytokine (e.g., IL-4, IL-5, and IL-13) production in BALF. In conclusion, SHL attenuates airway hyperresponsiveness and EAI mainly via the inhibition of mast cell activation and Th2 immunity, which may help to elucidate the underlying mechanism of SHL on asthma treatment and support its clinical use.

## 1. Introduction

Asthma is a chronic inflammatory disease of the airways. It affects approximately 300 million individuals of all age groups [1]. Owing to adult loss of productivity and child learning impairment, the disease has become a severe public health issue. Clinically, asthma is divided into allergic and nonallergic forms, which are distinguished by the presence or absence of clinical allergic reaction and* in vitro* IgE response to specific aeroallergens [2]. However, characteristic to both forms is the airway wall accumulation of activated Th2 cells, whose cytokines can drive infiltration of eosinophils [3]. Thus, asthma is classically thought to be positively controlled by Th2 cytokines. For example, IL-4 promotes IgE production by B cells [4], IL-5 causes development, recruitment, and activation of eosinophils [5], and IL-13 controls the effector phase of asthma by inducing airway hyperresponsiveness (AHR) and airway remodeling (AR), as well as hyperproduction of mucus [6].

In treatment, the actual basic strategy is to combine inhaled drugs that promote the rapid symptomatic relief of asthma exacerbation, reducing bronchoconstriction (long acting *β*2 agonists) and airway inflammation (corticosteroid). Many other medicines, such as anticholinergics, leukotriene modifiers, mast cell stabilizers, and Th2 cytokine antagonists, have also been used to control asthma [7]. Inhaled corticosteroids are considered as currently the most effective drugs available for the treatment of asthma [8]. However, currently there is no cure for asthma and long-term use of inhaled steroids is often accompanied by undesirable adverse effects, particularly when high doses are used [9, 10], which cry for highly active antiasthma drugs with no or lower side effects. In this context, herbal medicine which can harbor multiple components with a broad spectrum of anti-inflammatory activities has attracted a large attention.

Shuang-Huang-Lian (SHL), a commercial antimicrobial formulation comprising* Lonicera japonica, Scutellaria baicalensis*, and* Forsythia suspensa* is officially recorded in the Chinese Pharmacopoeia [11]. Clinically, SHL is not only used for the treatment of acute upper respiratory tract infection, acute bronchitis, and light pneumonia caused by bacteria/viruses [12], but also applied to treat bronchial asthma by ameliorating symptoms of respiratory tract, reducing dyspnea, and shortening the clinical course of the illness with intravenous infusion [13] or ultrasonic nebulization [14] for a long-term therapy. Moreover, our previous study revealed that SHL prevented basophil activation to suppress Th2 immunity [15] and stabilized mast cells through activation of mitochondrial calcium uniporter [16]. In addition, various pharmaceutical components in SHL exert an inhibitory effect on atopic asthma, such as chlorogenic acid [17], baicalin [18], and forsythiaside A [19], highly suggesting that SHL might possess the potential ability to treat bronchial asthma, especially for atopic asthma. However, to date, no studies have focused on the antiasthmatic properties of SHL. In this study, we investigated the protective effects of SHL on asthmatic responses using a shrimp protein (SP)- induced murine asthma model.

## 2. Materials and Methods

### 2.1. Materials

SHL lyophilized powder for injection was provided by Hayao Pharmaceutical Co., Ltd. (Harbin, Heilongjiang, China). Mouse IL-4, IL-5, eotaxin, and total IgE (tIgE) ELISA kits were from Biolegend Co. (San Diego, CA, USA). Mouse IL-13 ELISA kit was obtained from Excell Technology Co. (Shanghai, China). Mouse mast cell protease-1 (mMCP-1) ELISA kit was purchased from Thermo Fisher Scientific (CA, USA). Aluminium hydroxide gel was from Chemtrade LLC. (Berkeley, CA, USA). Methacholine (Mch) was obtained from Sigma-Aldrich (St. Louis, MO, USA).* O*-phenylenediamine-dihydrochloride (OPD) was from Tokyo Chemical Industry Co. Ltd. (Tokyo, Japan). Protein G PLUS-Agarose was obtained from Santa Cruz Biotechnology (CA, USA). The HRP-labeled rat anti-mouse IgE antibody was from Abcam Co. (Cambridge, UK). All other reagents were of analytical grade.

### 2.2. Animals

The Balb/c mice (female, 18 g-20 g) were purchased from Vital River Experimental Animal Services (Beijing, China) and housed in a SPF laboratory under standard temperature (22°C-24°C) and humidity (45% - 65%) conditions with a 12 h light/dark cycle and standard pallet diet and water ad libitum. All the animal experiments were carried out according to the National Institutes of Health Guide for Care and Use of Laboratory Animals and approved by the Institutional Animal Care and Use Committee (IACUC), Institute of Medicinal Plant Development (IMPLAD) of Chinese Academy of Medical Sciences (CAMS). Anesthetic drugs and all other necessary measures were used to reduce animal suffering during experimental procedures.

### 2.3. Preparation of SP

**Figure 1:**
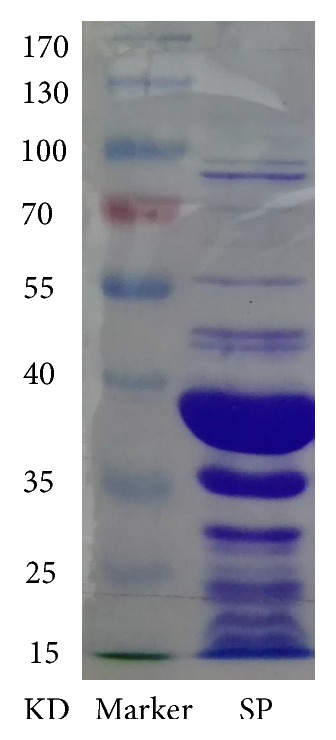
Electrophoretogram of SP by SDS-PAGE.

SP was prepared as previously described with some modifications [20]. Shrimp meat from vivid* Metapenaeus ensis* was ground and immersed in 0.1 M PBS (pH 7.4). After homogenizing and stirring, the shrimp homogenate stayed overnight at 4°C. The extracted solution was centrifuged (8,500 × g, 10 min, 4°C) and the supernatant was pooled for further purification. Grated (NH_4_)_2_SO_4_ was added to the supernatant up to the 60% salt concentration. 30 min later, the mixture was centrifuged (8, 500 × g, 5 min, 4°C). The supernatant was continuously added (NH_4_)_2_SO_4_ until the 90% salt concentration and centrifugation (16, 000 × g, 20 min, 4°C). The precipitation was dissolved in 0.01 M PBS (pH 7.4) and dialyzed in distilled water for 24 h. The extracted SP was then lyophilized and stored at −20°C for further use. The obtained SP was assayed by SDS-PAGE (Figure 1) and the extraction yield was 1.01%.

### 2.4. Induction of Allergic Asthma and Drug Intervention

**Figure 2:**
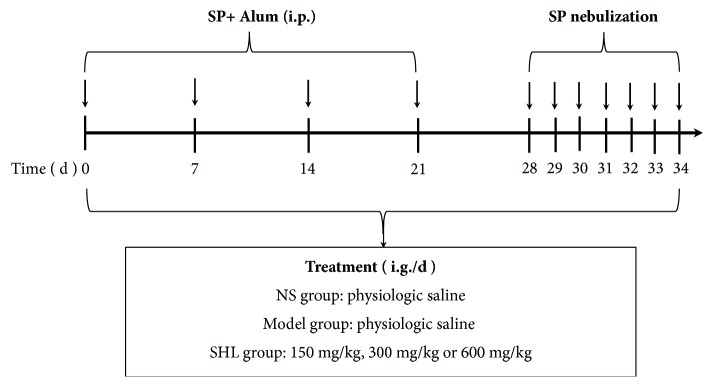
Protocol for the induction of murine asthma model and SHL treatment.

All mice were randomly separated into different groups (8 mice in each group) as follow: normal control group (normal saline, NS), model group (SP alone), and SHL groups (SP + SHL). Sensitization, nebulization, and treatment protocols for the different groups in this study were summarized in Figure 2 [21]. Mice were sensitized on day 0, 7, 14, and 21 by intraperitoneal injection of 60 *μ*g of SP with equal volume of aluminium hydroxide gel. Normal control mice received the same volume of NS and aluminium hydroxide gel. One week after the last sensitization, the mice were inhaled with SP (1 mg/mL, 8 mL) in an exposure chamber to challenge their airways with a nebulizer (PARI BOY SX, PARI GmbH, Starnberg, German) for 30 min on 7 consecutive days. The mice in SHL groups were daily treated (i.g.) with 150 mg/kg, 300 mg/kg, and 600 mg/kg of SHL dissolved in purified water, respectively.

### 2.5. Measurement of AHR

As an indicator of lung function, AHR was tested 24 h after the final nebulization using an AniRes 2005 Lung Function system (Bestlab 2.0, Beijing, China) according to manufacturer instructions. Mice were anaesthetized by pentobarbital sodium (i.p.). A connection was made by a computer-controlled ventilator via a cannula that had been implanted surgically in the trachea. The respiratory rate and the time ratio of expiration/inspiration were preset at 90/min and 2:1, respectively. When the parameters were stable, an injector needle was inserted into the jugular vein and Mch was administered through a catheter with increasing dosages of 0.025, 0.05, 0.1, and 0.2 mg/kg every 5 min [22]. Airway responsiveness was assessed by the peak value of inspiration resistance (R_L_) and expiration resistance (R_e_) after each injection [23].

### 2.6. Measurement of tIgE and SP-Specific IgE (sIgE)

Mice tail vein blood samples were collected and kept overnight at 4°C. tIgE levels in serum and bronchoalveolar lavage fluid (BALF) were measured by using an ELISA kit. The level of sIgE was measured as previously described [15].

### 2.7. Analysis of BALF Samples

Followed by the collection of blood samples, tracheotomy was performed. Airway lumina was washed with three successive ice-cold PBS (0.4 mL). BALF (1 mL) was collected and centrifuged (400 × g, 10 min, 4°C). The supernatant was used for cytokine analysis (IL-4, IL-5, IL-13, eotaxin, tIgE, and mMCP-1). Cell pellets were resuspended in PBS and total leukocytes were counted. Cell slides were prepared by a cytospin machine (2-16PK, Sartorius, Gottingen, Germany) and stained using Wright's buffer according to the manufacturer's instructions. Two hundred cells were counted per slide and the percentage of eosinophils was calculated [24].

### 2.8. Measurement of Eosinophil Peroxidase (EPO) Level

100 *μ*L of the substrate solution (1.5 mM OPD and 6.6 mM hydrogen peroxide in 0.05 M Tris-HCl, pH 8.0) was added to 100 *μ*L of sample in microtiter plates and incubated for 30 min at 37°C. The reaction was stopped by adding 50 *μ*L of 2 M sulfuric acid, and the optical densities (OD) were read at 492 nm [25, 26].

### 2.9. Restimulation of Mediastinal Lymph Nodes (MLNs) Cells with SP

The mice were sensitized (i.p., SP + Alum) for 4 weeks and inhaled SP aqueous solution for 5 days. Three days later, the mice were euthanized by cervical dislocation. MLNs were aseptically collected and made into single cell suspension by filtration through a 200 mesh cell strainer. Seed a round bottom 96-well culture plate with 2 × 10^5^ cells/well. MLNs cells were stimulated with SP (10 *μ*g/mL)* in vitro* and SHL treatment was performing at 0.15, 0.3, 0.6 mg/mL. Incubate the plate for 72 h in a humidified incubator with 5% CO_2_ at 37°C. The culture supernatant was collected for cytokine analysis (IL-4, IL-5, and IL-13) [27, 28].

### 2.10. Histopathological Examination

Left diaphragmatic lobe of lung and trachea were removed and placed into 4% paraformaldehyde (pH 7.4). For histopathological examination, 4 *μ*m sections of fixed embedded tissues were cut on a Leica rotary microtome, placed on glass slides, and deparaffinized. To determine the structural changes in airways, we used hematoxylin and eosin stain (HE) on sections of the lung tissue [29]. Histological analysis was performed by a light microscope.

### 2.11. Statistical Analysis

All data were expressed as mean ± standard deviation (SD). One-way analysis of variance was used to detect significant differences between the control and treatment groups. Student's* t*-test was used for multiple comparisons. Values of* P* < 0.05 were considered to be statistically significant.

## 3. Results

### 3.1. SHL Ameliorates AHR in the Murine Asthma Model

**Figure 3:**
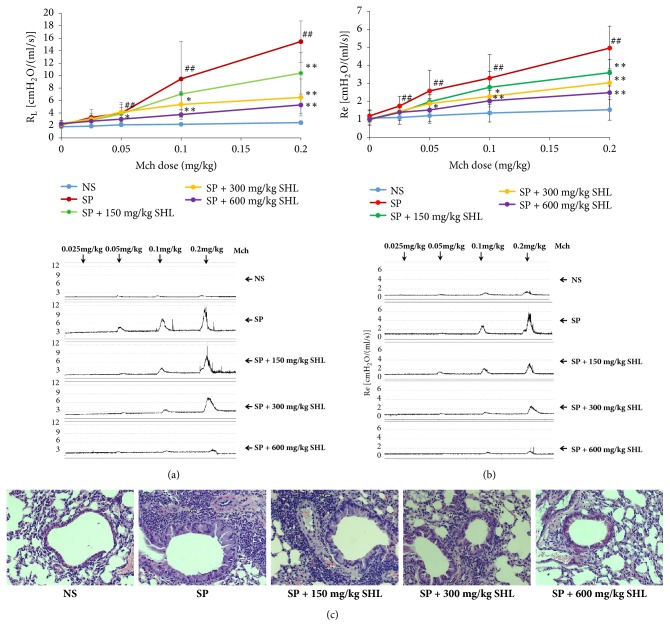
SHL prevents SP-induced AHR in the murine asthma model. (a-b) SHL prevents SP-induced AHR by comparing R_L_ (a) and Re (b) values with NS mice. 24 h after the last nebulization of SP, the mice were treated (i.v.) with Mch at the indicated doses and R_L_ and R_e_ values were recorded by AniRes 2005 mouse lung function analysis system. (c) Representative histological images of AR in lung tissue. Values were expressed as mean ± SD (n = 8). ^##^*P *< 0.01 versus NS; ^*∗*^*P* < 0.05 and ^*∗∗*^*P* < 0.01 versus SP.

AHR was tested by measuring airway resistance in response to Mch ranging from 0.025 mg/kg to 0.2 mg/kg 24 h after the last nebulization of SP. As shown in Figures 3(a) and 3(b), the model mice showed potent AHR in response to Mch. Although single-used (i.p.) SHL could not directly attenuate Mch (0.2 mg/kg)-induced AHR in normal mice (data not shown), continuous treatment with SHL could lead to a significant decrease in the AHR of model mice by comparing the R_L_ and R_e_ values with that of NS mice, suggesting that the ameliorative effect of SHL on SP-induced AHR was not attributed to a directly anticholinergic activity.

We next investigated whether SHL could affect SP-induced AR. As shown in Figure 3(c), SP contributed to a hypertrophy of mucous epithelium cells and thickness of smooth muscle. Treatment with SHL ameliorated SP-induced hypertrophy of mucous epithelium cells and reduced the thickness of smooth muscle, suggesting an ameliorative effect of SHL on AR.

### 3.2. SHL Attenuates Eosinophilic Airway Inflammation (EAI) in the SP-Induced Asthmatic Mice

**Figure 4:**
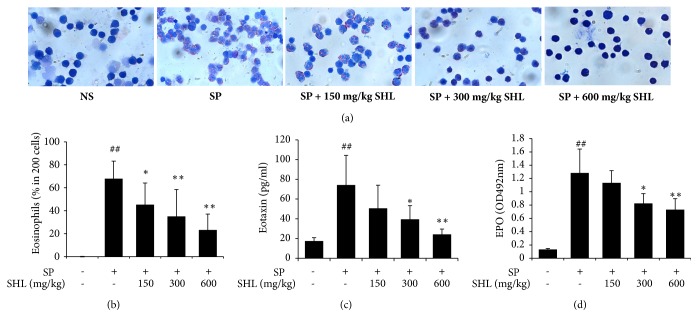
Effect of SHL on SP-induced EAI. (a) Representative images of eosinophil infiltration in BALF. The mice were sacrificed and the BALF was collected. Cytospin preparations were made for the leukocytes differential cell count by Wright's staining. (b) The proportion of eosinophils in leukocytes in BALF. (c-d) SHL reduced eotaxin and EPO levels in BALF. Values were expressed as mean ± SD (n = 8). ^##^*P *< 0.01 versus NS; ^*∗*^*P* < 0.05 and ^*∗∗*^*P* < 0.01 versus SP.

One of the characteristics of asthma is the infiltration of inflammatory cells, especially eosinophils, into the airway mucosa, where eosinophils act as effector cells by releasing cytotoxic granule proteins and then lead to EAI [30]. Therefore, we collected the BALF from the mice 24 h after the last SP nebulization to investigate the effect of SHL on EAI. The number of eosinophils was counted according to their bilobed nuclei and large acidophilic granules in cytoplasm. As shown in Figures 4(a) and 4(b), SP dramatically increased the proportion of eosinophils, while SHL potently reduced SP-elevated eosinophils in a dose-dependent manner (Figure 4(b)). Next we determined the levels of eotaxin (a potent chemoattractant for eosinophils) [31] and EPO (one of the cytotoxic molecules released by activated eosinophils) [32]. The obtained results showed that SP markedly elevated eotaxin and EPO levels, while SHL significantly decreased eotaxin and EPO at the dose of 300 mg/kg (Figure 4(c)) and 600 mg/kg (Figure 4(d)), showing an inhibitory effect on the SP-induced EAI.

### 3.3. SHL Suppresses SP-Elevated IgE and mMCP-1 Levels

**Figure 5:**
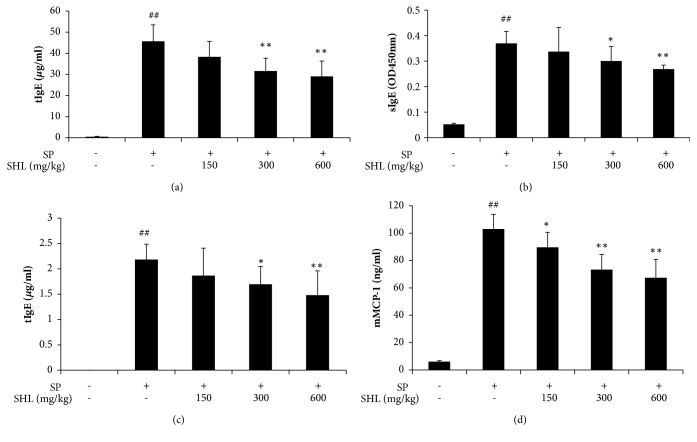
Effects of SHL on SP-elevated IgE and mMCP-1 levels. (a-b) SHL reduced tIgE and sIgE in serum. (c-d) SHL decreased tIgE and mMCP-1 levels in BALF. Values were expressed as mean ± SD (n = 8). ^##^*P *< 0.01 versus NS; ^*∗*^*P* < 0.05 and ^*∗∗*^*P* < 0.01 versus SP.

IgE-mediated degranulation of mast cells results in acute allergic symptoms and recruitment of eosinophils to the site of inflammation [33, 34]. We next determined the IgE level in serum and BALF. As shown in Figures 5(a)–5(c), SP caused a significant increase of tIgE, while SHL markedly decreased SP-elevated tIgE. Meanwhile, given that sIgE was enough to be detected after removing IgG from serum, we measured its concentration. Similar to tIgE, sIgE increased significantly after SP stimulation and SHL lowered SP-elevated sIgE (Figure 5(b)).

mMCP-1, released by the activated mast cells, is a specific marker of IgE-mediated anaphylaxis [35]. SP could cause a considerable rise in mMCP-1, while SHL significantly lowered the mMCP-1 level (Figure 5(d)), indicating that SHL could inhibit mast cell activation in type I hypersensitivity.

### 3.4. SHL Decreases Th2 Cytokines in BALF and MLNs Cells

**Figure 6:**
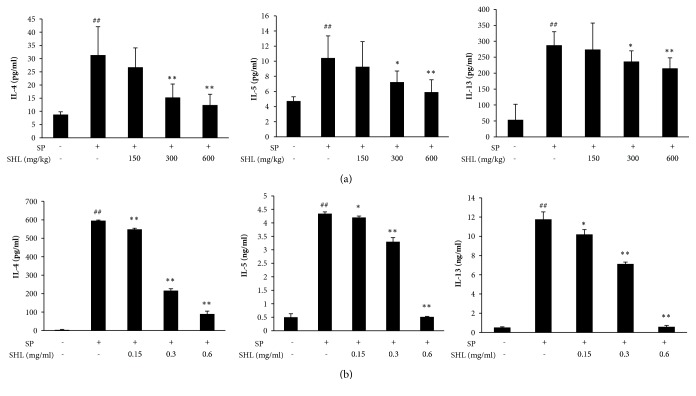
Effect of SHL on Th2 cytokines in BALF and their production in MLNs cells. (a) SHL decreased IL-4, IL-5, and IL-13 levels in BALF. (b) SHL decreased IL-4, IL-5, and IL-13 production in MLNs cells. Three days after the last inhalation of SP, mice were sacrificed and the MLNs were isolated. Single cell suspensions were aseptically prepared and seeded in a round bottom 96-well plate (2 × 10^6^ cells/well) and then stimulated with SP (10 *μ*g/mL) at 37°C for 72 h. The levels of IL-4, IL-5, and IL-13 in the culture medium were measured using the ELISA kits. Values were expressed as mean ± SD (n = 8). ^##^*P *< 0.01 versus NS; ^*∗*^*P* < 0.05 and ^*∗∗*^*P* < 0.01 versus SP.

Asthma is classically thought to be a Th2-cell driven complex inflammatory disease [36]. Therefore, we next determined the effect of SHL on Th2 cytokine production in response to SP in BALF of sensitized mice. As shown in Figure 6(a), SP dramatically elevated IL-4, IL-5, and IL-13 levels, while SHL significantly suppressed the production of these Th2 cytokines in a dose-dependent manner.

Because the Th2 immunity in the lung tissue mainly originates from the draining MLNs [37], we tested the effect of SHL on Th2 cytokines released by the MLNs cells from SP-sensitized mice. The obtained results showed that SP could potently activate its specific Th2 cells in MLNs, while SHL markedly suppressed the activated-Th2 cells to release IL-4, IL-5, and IL-13 in a concentration-dependent manner (Figure 6(b)) without cytotoxicity (Figure S1).

## 4. Discussion

In the food allergen-induced asthma models, ovalbumin (OVA) is a well-established commonly used allergen. But shrimp, a familiar edible shellfish, might induce more wide-ranging allergic asthma in contrast to egg [38, 39]. As we know, tropomyosin is the major allergen of shellfish, and IgE cross-reactivity between various shellfish species or between shellfish and other invertebrates is a common phenomenon [40]. Clinically, among subjects sensitized to allergens containing tropomyosin, the proportion of coexisting sensitization to shellfish, cockroach, and dust mite was quite high [41]. Thus, shrimp-based asthma model should be more representative compared with OVA. In the present study, we deliberately chose SP as the allergen to establish a murine asthma model.

Asthma is a disorder of the conducting airways leading to variable airflow obstruction whose principal causes are AR and AHR [42, 43]. Airway inflammation is central to asthmatic pathophysiology and leads to AR, characterized by mucus hypersecretion, epithelial fibrosis, metaplasia and hyperplasia of goblet cells, and airway smooth muscle hypertrophy and hyperplasia [44]. In clinical, SHL has been used to treat bronchial asthma [13, 45]. Consistently, our data demonstrated that SHL inhibited airway inspiratory and expiratory resistance in SP-induced asthmatic mice (Figures 3(a) and 3(b)), and histological studies suggested it reduced the thickness of airway smooth muscle and the infiltration of inflammation cells (Figure 3(c)).

EAI, characterized by recruiting eosinophils to bronchus and activating them, is a predominant airway inflammation in asthma [46]. Our data clearly showed that SP markedly induced the intense infiltration of eosinophils around bronchus (Figure 3(c)) and increased the relative proportion of eosinophils in BALF (Figure 4(a)). As a marker of eosinophil activation, EPO was also significantly elevated in BALF after SP stimulation (Figure 4(d)), displaying the feature of EAI. To our knowledge, eosinophils can be recruited and activated by multifactors, such as Th2 cytokines (e.g., IL-4, IL-5, and IL-13) [47], eotaxin [48], and various bioactive substances produced in the process of mast cells degranulation (e.g., mMCP-1) [49]. SHL not only significantly suppressed SP-elevated Th2 cytokine production, decreased BALF eotaxin and mMCP-1 levels (Figures 4(c), 5(d), and 6(a)), but also reduced the recruitment and activation of eosinophils (Figures 3(c) and 4).

Asthma can be divided into atopic and nonatopic phenotypes, which share common features of lung hypersensitivity and are classically thought to be a Th2-cell driven complex inflammatory disease. Atopic asthma is mediated by IgE and is usually caused by allergens [36]. After antigen stimulation, basophils are transient recruited into draining lymph nodes to drive Th2 cell differentiation, thus promoting sIgE production by B cells. The produced sIgE can sensitize peribronchial mast cells through binding to their high affinity IgE receptor (Fc*ε*RI) [50]. Under re-exposure of the antigen, the sensitized mast cells are activated and subsequently release inflammatory cytokines and chemokines, as well as mediators of airway spasm and hyperreactivity [51]. In the present study, we found that after SP systemic sensitization and atomization challenge, the SP-specific Th2 cells appeared in MLNs (Figure 6(b)). Moreover, IgE levels, including tIgE and sIgE, were dramatically increased in serum and/or BALF (Figures 5(a)–5(c)), showing an obvious characteristic of atopic asthma. SHL significantly suppressed SP-elevated tIgE levels in serum and BALF (Figures 5(a)–5(c)), decreased serum sIgE (Figure 5(b)), and inhibited the ability of SP-activated Th2 cells in MLNs to release IL-4, IL-5, and IL-13 (Figure 6(b)).

In summary, our findings reveal, for the first time, that SHL alleviates SP-induced AHR and EAI in a murine asthma model, mainly through inhibiting mast cell activation and Th2 immunity. The latter mechanism probably involves in preventing Th2 differentiation in MLNs and suppressing Th2 cytokines in lung tissue, thus lowering IgE level, decreasing eotaxin and EPO levels, and reducing the recruitment of eosinophils. Our study provides a pharmacological basis for the clinical use of SHL to relieve bronchial asthma.

## Data Availability

The datasets used and/or analyzed during the current study are available from the corresponding author on reasonable request.

## Supplementary Materials

Supplementary Materials FIGURE S1. Effect of SHL on the viability of MLNs cells. Data are expressed as mean ± SD (n = 3).
