# Supplementary material for: Shuang-Huang-Lian Attenuates Airway Hyperresponsiveness and Inflammation in a Shrimp Protein-Induced Murine Asthma Model
Source: Evid Based Complement Alternat Med. 2019 Jan 1;2019:4827342. doi: 10.1155/2019/4827342 (PMC6332955; doi:10.1155/2019/4827342)

Supplementary Materials

Fig S1. Effect of SHL on the viability of MLN cells. Data are expressed as mean ± SD (n = 3).

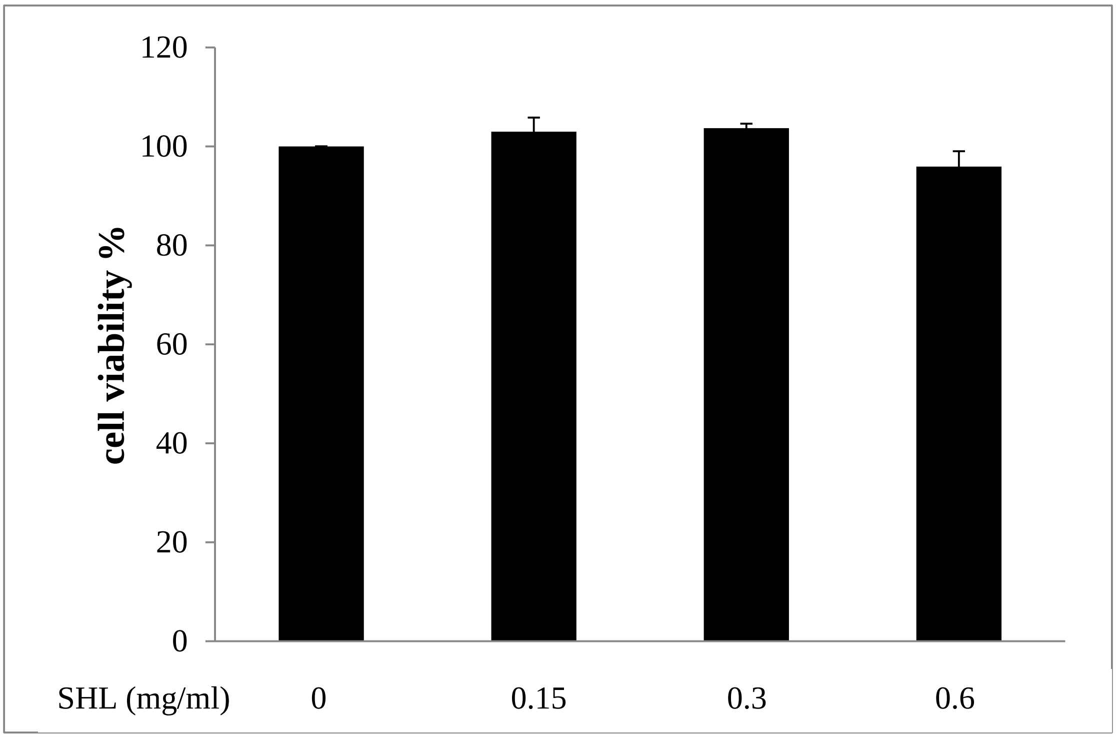

Supplement: Supplementary Materials — FIGURE S1. Effect of SHL on the viability of MLNs cells. Data are expressed as mean ± SD (n = 3). [file 4827342.f1.pdf]
